# Curcumin-Gene Expression Response in Hormone Dependent and Independent Metastatic Prostate Cancer Cells

**DOI:** 10.3390/ijms20194891

**Published:** 2019-10-02

**Authors:** Shilpa Katta, Arun Srivastava, Rajesh L. Thangapazham, Inger L. Rosner, Jennifer Cullen, Hua Li, Shashwat Sharad

**Affiliations:** 1 Center for Prostate Disease Research, Department of Surgery, Uniformed Services University of the Health Sciences and the Walter Reed National Military Medical Center, 6720A Rockledge Dr., Suite 300, Bethesda, MD 20817, USA; shilpa.katta@nih.gov (S.K.); aruns1207@gmail.com (A.S.); rajeshlt@gmail.com (R.L.T.); inger.l.rosner.mil@mail.mil (I.L.R.); jcullen@cpdr.org (J.C.); 2 Henry Jackson Foundation for the Advancement of Military Medicine, 6720A Rockledge Dr., Suite 300, Bethesda, MD 20817, USA; 3 Department of Urology, Walter Reed National Military Medical Center, 8901 Rockville Pike, Bethesda, MD 20889, USA

**Keywords:** curcumin, prostate cancer, TGF-β, MYC, AR, chemotherapy, metastasis, signaling pathways

## Abstract

The androgen receptor is one of the key targets for prostate cancer treatment. Despite its less satisfactory effects, chemotherapy is the most common treatment option for metastatic and/or castration-resistant patients. There are constant needs for novel anti-prostate cancer therapeutic/prevention agents. Curcumin, a known chemo-preventive agent, was shown to inhibit prostate cancer cell growth. This study aimed to unravel the inhibitory effect of curcumin in prostate cancer through analyzing the alterations of expressions of curcumin targeting genes clusters in androgen-dependent LNCaP cells and androgen-independent metastatic C4-2B cells. Hierarchical clustering showed the highest number of differentially expressed genes at 12 h post treatment in both cells, suggesting that the androgen-dependent/independent manner of curcumin impacts on prostate cancer cells. Evaluation of significantly regulated top canonical pathways highlighted that Transforming growth factor beta (TGF-β), Wingless-related integration site (Wnt), Phosphoinositide 3-kinase/Protein Kinase B/ mammalian target of rapamycin (PIK3/AKT(PKB)/mTOR), and nuclear factor kappa-light-chain-enhancer of activated B cells (NF-kB) signaling were primarily inhibited, and Phosphatase and tensin homolog (PTEN) dependent cell cycle arrest and apoptosis pathways were elevated with curcumin treatment. The short term (3–24 h) and long term (48 h) effect of curcumin treatment revealed 31 and four genes modulated in both cell lines. TGF-β signaling, including the androgen/TGF-β inhibitor Prostate transmembrane protein androgen-induced 1 (*PMEPA1*), was the only pathway impacted by curcumin treatment after 48 h. Our findings also established that *MYC* Proto-Oncogene, basic helix-loop-helix (bHLH) Transcription Factor (MYC) signaling was down-regulated in curcumin-treated cell lines. This study established, for the first time, novel gene-networks and signaling pathways confirming the chemo-preventive and cancer-growth inhibitory nature of curcumin as a natural anti-prostate cancer compound.

## 1. Introduction

Prostate cancer is the second most frequent male cancer and a leading cause of morbidity and mortality in men worldwide [1]. Despite advances in cancer therapy, a significant decrease in the incidence and mortality rates of prostate cancer has not been observed in the past several years [2]. Currently, there have been no widely accepted medications to reduce the risk of prostate cancer progression with active surveillance. The need for novel and/or modification of known therapeutic agents against prostate cancer has constantly been warranted. For prostate cancer prevention and treatment, it has been very important to dissect the underlying molecular signaling pathways contributing to cancer development and progression. Epidemiological studies have identified that race, age, family history, diet—including eating habits—and lifestyle were all prominent risk factors for prostate cancer [3,4]. Since prostate cancer tended to develop in later age (>50 years), diet modification in an earlier age might decrease disease incidence. Nutraceuticals have been shown to be one of the promising sources of therapy for the prevention and treatment of various human diseases. Curcuma longa (curcumin) is an extensively characterized nutraceutical extracted from the turmeric plant in a pure crystalline form [5]. Curcumin’s anti-tumor, antioxidant, and anti-inflammatory properties were discovered decades ago [6]. In India, turmeric is commonly used in Indian cooking as well as a traditional anti-inflammatory medicine to accelerate wound healing; curcumin is a major effective anti-inflammatory component [5,6]. Curcumin has been shown to inhibit proliferation and invasion and induce apoptosis of prostate cancer cells in vitro and in vivo through interfering with various signaling pathways including mitogen-activated protein kinase (MAPK), epidermal growth factor receptor (EGFR), and nuclear factor κ (NFκB) [7,8,9]. The NF-kB transcription factor plays a major role in the regulation of genes involved in inflammation, cell proliferation, and cell survival. Curcumin has been reported to directly bind and down-regulate many NF-kB regulated genes such as *COX-2* (Cyclooxygenase-2), *5-LOX* (5-lipoxygenase), *TNF* (Tumor necrosis factor), *IL-6* (Interleukin 6), as well as inhibit *EGFR* tyrosine kinase activity [10,11,12,13,14]. Androgens play an important role in the development and progression of prostate cancer by binding to androgen receptors (ARs), a member of the steroid receptor family [15,16,17]. Mutations and amplifications of AR lead to abnormal activation of androgen signaling to facilitate prostate cancer aggressive progression [18]. Curcumin was revealed to suppress the expression of ARs and AR-associated cofactors [19,20]. Our previous study has shown that curcumin caused the decrease in expression of various AR regulated genes (*NKX3.1* (NK3 Homeobox 1), *KLK3*/(PSA) (Kallikrein related peptidase 3/ Prostate-specific antigen), *TMPRSS2* (Transmembrane serine protease 2) in a time-dependent manner in both androgen-dependent LNCaP and androgen-independent C4-2B cells [21]. Cancer is a hyperproliferative disease, and nearly 90% of cancer-associated deaths are due to metastasis [22]. It is well understood that the prostate cancer progression and bone metastasis is mediated through dysregulation of multiple cell signaling pathways, and the majority of prostate cancer drugs control specific targets. 

The goal of this proof-of-concept study is to carefully evaluate the comparative gene expression signature of LNCaP and C4-2B prostate cancer cell lines after curcumin treatment. In this study, we extended our knowledge to localize new gene signatures and signaling pathways responding to curcumin treatment in prostate cancer cells to further elucidate the anti-tumor mechanism of curcumin. This study highlights the long- and short-term effect of curcumin treatment on multiple signaling pathways linked to prostate cancer progression and metastasis in the androgen-dependent and independent stages. These data will provide the foundation for targeted studies focusing on molecular mechanisms of prostate cancer prevention and treatment. 

## 2. Results

### 2.1. Gene Expression Responses to Curcumin in Androgen-Dependent LNCaP Cells and Androgen-Independent Metastatic Prostate Cancer C4-2B Cells 

The androgen-dependent LNCaP cells and androgen-independent metastatic prostate cancer cells C4-2B were treated with 10 μM of curcumin for 3, 6, 12, 24 and 48 h. Androgen responsive features of LNCaP and androgen-refractory features of C4-2B cells were capitalized to identify the curcumin response in metastatic androgen inhibition sensitive (C4-2B) and less aggressive (LNCaP) tumor cell lines. Microarray results from time-course dependent treatment of curcumin in prostate cancer cell lines were analyzed. Pair-wise comparisons were performed on the datasets to identify differentially expressed genes. The comparison ratios were calculated by dividing the gene expressions of the curcumin-treated cells with untreated control cells at different time points. To further identify genes with statistically significant alterations, an arbitrary two-fold cut-off of the changes of transcript levels of impacted genes was applied. Our data revealed that multiple genes were impacted by curcumin treatment with differential expressions. Based on gene expression profiles over the time course, we identified the most up- and down-regulated genes by curcumin treatment (Table 1). Further, it was noted that 12 h post curcumin treatment was the peak time point with maximum number of genes affected by curcumin treatment in both LNCaP (1273 genes up-regulated and 1682 genes down-regulated) and C4-2B (1119 genes up-regulated and 943 genes down-regulated) cells. The number of genes modulated by curcumin was more prominent in the LNCaP cells than C4-2B cells. It was further found that the expression of most impacted genes returned to normal levels within 24 to 48 h post treatment.

### 2.2. Defining the Prostate Cancer Signature of Differentially Regulated Genes Common in Both LNCaP and C4-2B Cells Post-Curcumin Treatment

**Figure 1:**
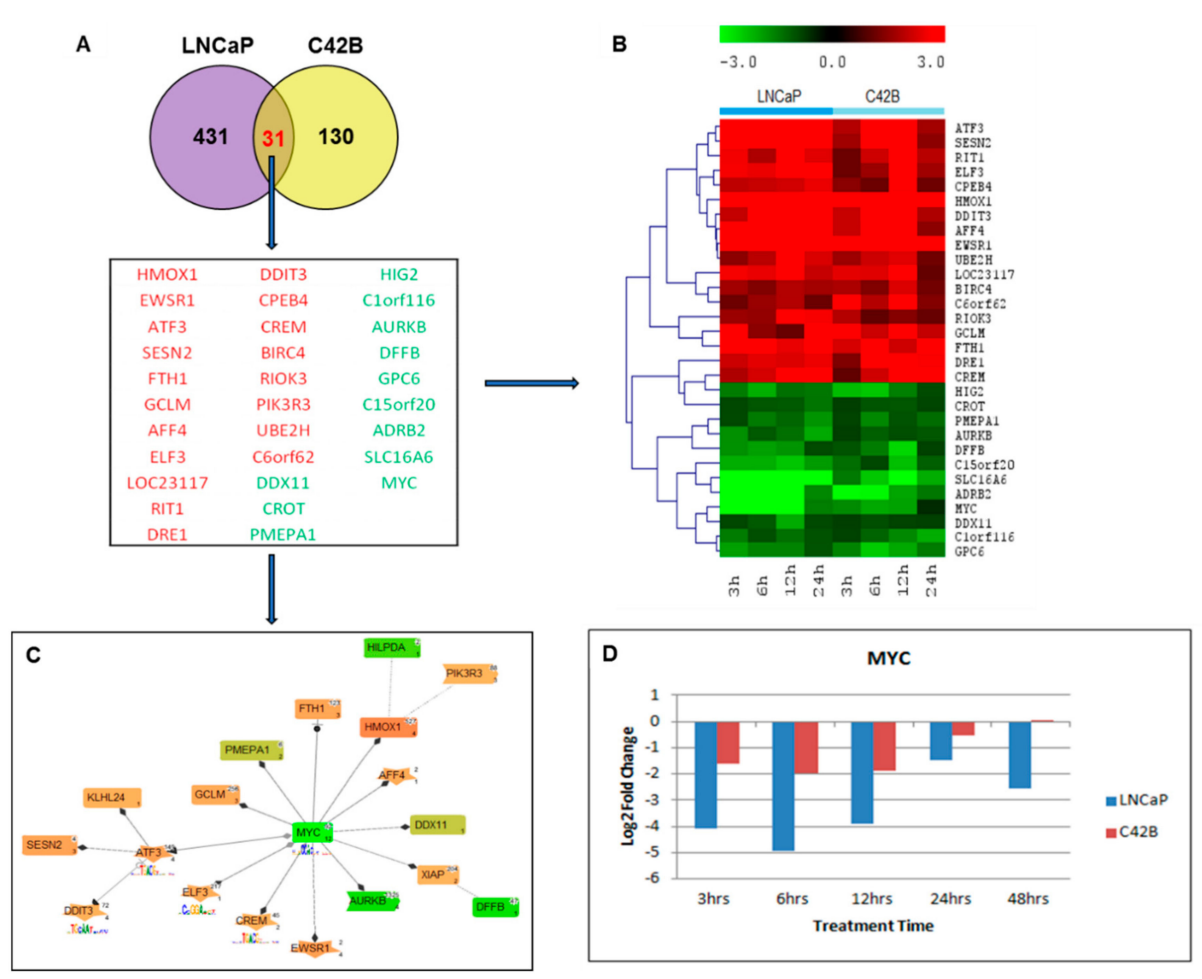
Functional analysis of differentially expressed genes between LNCaP and C4-2B cells by GePS system. (**A**) The total of 462 and 161 differentially expressed genes were identified from 3–24 h time points in LNCaP and C4-2B cells, respectively. Only 31 genes were found to be co-differentially expressed in both the cell lines. The Venn diagram analysis represents the overlap of LNCaP (purple) and C4-2B (yellow) significant genes with at least 3-fold expression. (**B**) Heatmap with hierarchical cluster showing the expression levels of the 31 significant genes shared between LNCaP and C4-2B cells clustered into up-regulated (shaded red) and down-regulated (shaded green) groups. (**C**) The 31 LNCaP and C4-2B shared genes were imported into GePS. Orange/red color shows up-regulation and green color shows down-regulation. The intensity of green and red colors indicates the degree of up or down-regulation, respectively. A solid line represents an expert-curated association between the two gene products, and a dotted line means there is an association by co-citation. This figure illustrates *MYC* as a significant gene in a central position with the most connected nodes. (**D**) Time course fold change values (log2) of *MYC* in LNCaP and C4-2B cell lines after curcumin treatment.

First, we focused on the impacted genes by short-term effects of curcumin treatment in both LNCaP and C4-2B cells. Here, we defined the time points of 3, 6, 12, and 24 h post curcumin treatment as short-term, based on the half-life of curcumin. The totals of 462 and 161 differentially regulated genes (DEGs) were identified after curcumin treatment with more stringent three-fold cut-off from 3–24 h in LNCaP and C4-2B cells, respectively (Figure 1A). The Genomatix Pathway system (GePS) analysis revealed 31 shared differentially expressed genes in LNCaP and C4-2B cells at 3 h, 6 h, 12 h, and 24 h time points (Figure 1A). The 19 genes coded in red color (Heme Oxygenase-1 (*HMOX1*), Ewing sarcoma breakpoint region 1 (*EWSR1*), Cyclic AMP-dependent transcription factor (*ATF3*), Sestrin-2 (*SESN2*), Ferritin Heavy Chain 1 (*FTH1*), Glutamate-Cysteine Ligase Modifier Subunit (*GCLM*), AF4/FMR2 Family Member 4 (*AFF4*), E74 Like ETS Transcription Factor 3 *(ELF3), RIT1, DRE1, DDIT3,* Cytoplasmic Polyadenylation Element Binding Protein 4 (*CPEB4*), CAMP Responsive Element Modulator (*CREM*), Bculoviral IAP repeat-containing protein 4 (*BIRC4*), RIO Kinase 3 (*RIOK3*), Phosphoinositide-3-Kinase Regulatory Subunit 3 (*PIK3R3*), Ubiquitin Conjugating Enzyme E2 H (*UBE2H*), and Chromosome 6 open reading frame 62 (*C6orf62*) were up-regulated, and 12 genes coded in green color DEAD/H-Box Helicase 11 (*DDX11),* Carnitine O-Octanoyltransferase *(CROT),* Prostate Transmembrane Protein, Androgen Induced 1 (*PMEPA1*), Hypoxia-inducible gene 2 (*HIG2*), Chromosome 1 Open Reading Frame 116 (*C1orf116*), Aurora Kinase B (*AURKB*), DNA Fragmentation Factor Subunit Beta (*DFFB*), Glypican-6 (*GPC6*), Chromosome 15 Open Reading Frame 20 (*C15orf20*), Adrenoceptor Beta 2 (*ADRB2*), Solute Carrier Family 16 Member 6 (*SLC16A6*), and *MYC* Proto-Oncogene, basic helix-loop-helix (bHLH) Transcription Factor (*MYC*) were down-regulated (Figure 1A). The heat map represents the expression levels of genes with up- or down- regulation at a −3.0 to 3.0 scale (Figure 1B). The signaling pathway networking analysis centering around 31 shared differentially expressed genes was further outlined, with the solid line representing an expert-curated association between the two gene products, and the dotted line standing for an association by co-citation. Next, to understand the molecular mechanisms involved in the response to curcumin treatment, we constructed a pathway/network Venn diagram analysis of all the differentially expressed genes with over 2-fold change by Genomatix Network and Pathway Analysis (GePS) System (Figure 1C). It was demonstrated that more genes in LNCaP were affected by Curcumin when compared to C4-2B cells (462 versus 161), and there were only 31 genes impacted in both the cells, accounting for around 5% of total genes studied. Network analysis further revealed that MYC as the common gene with most genetic interactions with other impacted genes in both the cells, which was down-regulated in curcumin-treated cells. The other two genes Heme Oxygenase-1 (*HMOX1*) and Cyclic AMP-dependent transcription factor (*ATF–3*), were found to be up-regulated in both cells. We first examined significant genes forming the central nodes based on the gene score (score represents numerous interactions of a gene). Genes with high numbers of interactions were searched in the literature with co-citation level (gene name localized at the abstract level) and were further filtered by literature free text search “prostate cancer” and “curcumin” after adding the total number of genes in the resulted network in LNCaP and C4-2B cells. The time course fold change (log2) analysis highlighted MYC as the most significantly down-regulated gene after curcumin treatment in both the LNCaP and C4-2B cell lines (Figure 1D).

### 2.3. Gene Ontology and Network Analysis of Differentially Expressed Genes Unique to Androgen Responsive Less Aggressive LNCaP Cells

**Figure 2:**
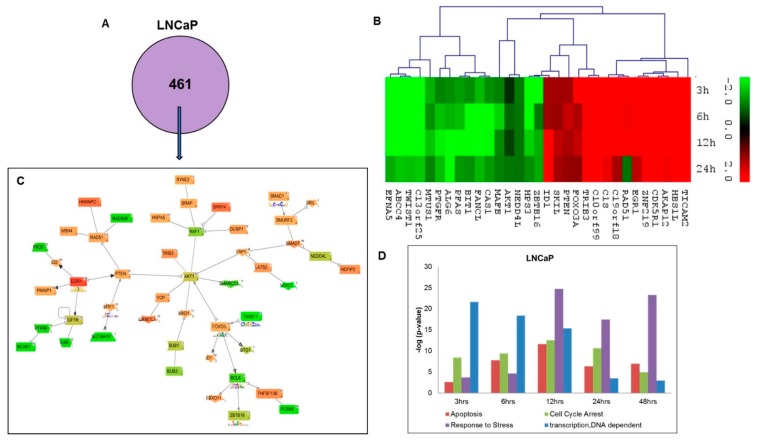
Functional analysis of differentially expressed genes unique to LNCaP cells after curcumin treatment. (**A**) GePS analysis of significant differentially regulated unique genes (431 genes) to LNCaP cells. (**B**) Heatmap of top up-regulated and down-regulated genes in LNCaP cells. The intensity of green and red colors indicates the degree of up or down-regulation, respectively. (**C**) Expert level filter settings were used to generate the network, which contains interactions curated by experts (Genomatix and NetPro) based on literature; genes without any interactions were filtered out. This figure illustrates *AKT1, PTEN,* and *EGR1* as significant genes in LNCaP with the most connected nodes. (**D**) Gene ontology analysis (Biological Processes) using Genomatix GeneRanker and DAVID Functional Annotation Tool of the unique gene lists from LNCaP cell lines. The majority of the genes’ activities involved in the above-mentioned annotations/processes were found to be increased till 12 h time point.

To identify a unique pathway/network of genes affected during the short-term time-course of curcumin treatment in LNCaP cells, all the differentially expressed genes at all the time points; 3 h, 6 h, 12 h and 24 h were uploaded into the Genomatix Network and Pathway Analysis (GePS) software for analysis. A total of 431 differentially expressed genes were found to be unique to LNCaP cells, but not affected in C4-2B cells (Figure 2A). The heat map summarized the expression levels of top 31 unique genes (top 15 up- and top 16 down-regulated) at –3.0 to 3.0 scales in LNCaP cells based on the cut-off of two-fold change (Figure 2B). To further dissect the impacted signaling pathways by curcumin treatment, we constructed the pathway/network system of all the differentially expressed genes unique to LNCaP cells with a cut-off of over 2-fold change (Figure 2C). Venn diagram analysis revealed that 9 central nodes, based on the gene score, formed (score represents the numerous interactions of a gene). The analysis further identified 3 down-regulated central nodes, including *RAF1* (Raf-1 Proto-Oncogene, Serine/Threonine Kinase), *BCL6* (B-Cell CLL/Lymphoma 6), *IGF1R* (Insulin-Like Growth Factor 1 Receptor), as well as 5 up-regulated central nodes such as *PTEN* (Phosphatase And Tensin Homolog), *EGFR1* (Epidermal Growth Factor Receptor 1), *SMAD7* (SMAD Family Member 7), *FOXO3* (Forkhead Box O3), *AKT1* (V-Akt Murine Thymoma Viral Oncogene Homolog 1) and *RAD51* (RAD51 Recombinase). For a more detailed pathway analysis, we used the gene score (score represents numerous interactions of a gene) to screen most affected genes forming 9 central nodes. The genes with the highest number of interaction hits were further researched as co-citation level and filtered by literature free text search “prostate cancer”, “curcumin”, and the genes presented in the gene network formed above. The signaling pathways of apoptosis, cell cycle arrest, stress response genes, and DNA dependent transcription were revealed as the most significantly affected pathways responding to curcumin treatment in LNCaP cells (Figure 2D). 

### 2.4. Gene Ontology and Network Analysis of Differentially Expressed Genes Unique to Androgen-Independent Highly Metastatic C4-2B Cells

**Figure 3:**
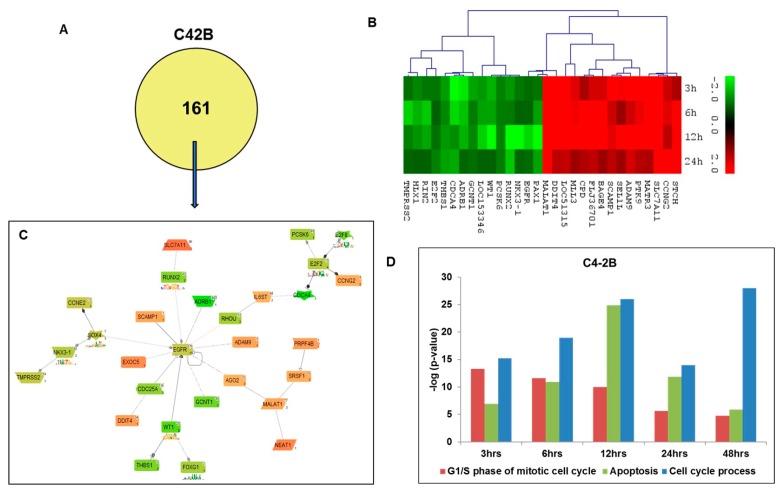
Functional analysis of differentially expressed genes unique to C4-2B cells after curcumin treatment. (**A**) GePS analysis of significant unique genes (130 genes) to C4-2B cells. (**B**) Heatmap of the top up-regulated and down-regulated genes in C4-2B. The intensity of green and red colors indicates the degree of up or down-regulation, respectively. (**C**) Expert level filter settings were used to generate the network and genes without any interactions were filtered out. The network was constructed from 130 input genes and illustrated *EGFR* and *SOX4* as significant genes in C4-2B with the most connected nodes. (**D**) Gene ontology analysis (Biological Processes)—using Genomatix GeneRanker and DAVID Functional Annotation Tool—of the unique gene lists from C4-2B cell lines. The majority of the genes’ activities involved mentioned above annotations/processes were found to be increased till 12 h time point.

Further, we analyzed the unique genes affected by curcumin treatment in C4-2B cells at short-term time points; 3, 6, 12 and 24 h similar to LNCaP cells. A total of 130 differentially expressed genes (Figure 3A) were found to be unique to C4-2B cells. The heat map summarizes the expression levels of the top 30 affected genes (top 15 up- and 15 down-regulated) at –3.0 to 3.0 scales in C4-2B cells based on a two-fold change of expression levels compared to no treatment control (Figure 3B). The pathways/networks of these 130 genes were further constructed by GePS software (Figure 3C). The pathway analysis represented an expert-curated association between the two affected genes, forming 5 central nodes based on the gene scores, revealing the four down-regulated central nodes, including *SOX4* (SRY (Sex Determining Region Y)-Box 4, *EGFR* (Epidermal Growth Factor Receptor), *WT1* (Wilms Tumor 1), *E2F2* (E2F Transcription Factor 2), and one up-regulated *MALAT1* (Metastasis Associated Lung Adenocarcinoma Transcript 1) central node in curcumin-treated C4-2B cells. Furthermore, pathway analysis revealed that the most impacted signaling pathways are the pathways associated with apoptosis, cell cycle processes, and the G1/S phase of the mitotic cell cycle in C4-2B cells (Figure 3D).

### 2.5. Canonical Pathway Analysis of Curcumin Response Genes in Both LNCaP and C4-2B Cells

**Figure 4:**
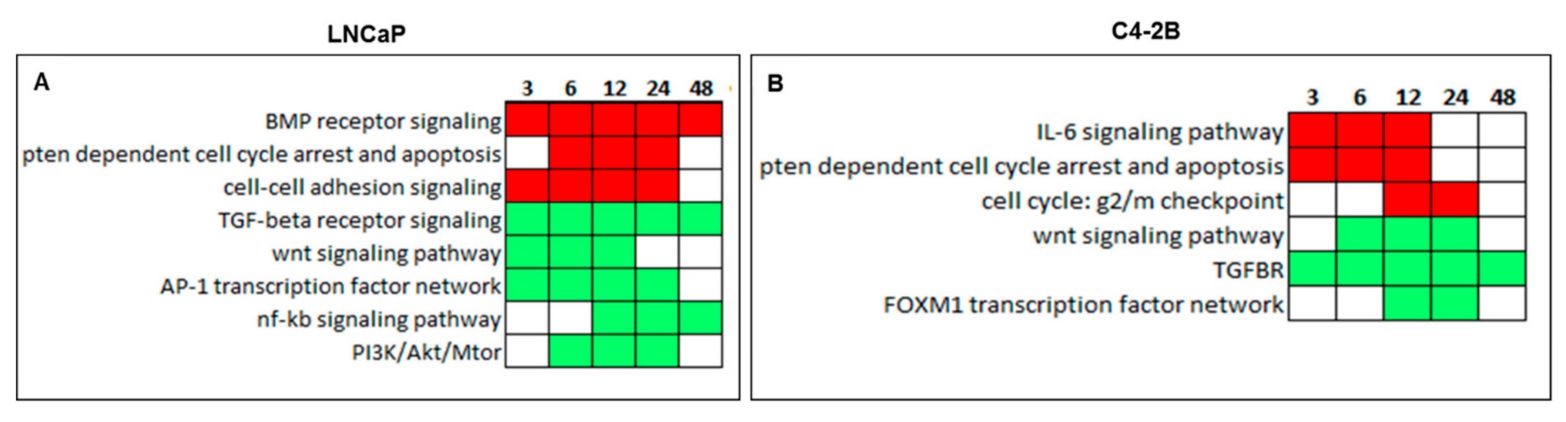
Heatmap of the top canonical pathway categories in LNCaP and C4-2B cells after curcumin treatment at 3–48 h time point. Canonical Pathway Analysis by Genomatix GeneRanker showed the long-term effect of curcumin treatment is only TGF-β receptor in both (**A**) LNCaP and (**B**) C4-2B cell lines at 48 h time point. Red squares are categories over-represented in up-regulated genes, and green squares are categories over-represented in down-regulated genes.

The Canonical Pathway Analysis of curcumin response genes was done by Genomatix GeneRanker software in both LNCaP and C4-2B cells. Top Canonical Pathways significantly altered by curcumin treatment over the 3–48 h time course in LNCaP and C4-2B cells are represented as a heat map in Figure 4. Red squares are signaling pathways represented by up-regulated genes, and green squares are the ones represented by down-regulated genes. In less aggressive LNCaP cells, BMP receptor signaling, PTEN-dependent cell cycle arrest, apoptosis, and cell-cell adhesion signaling pathways were found to be up-regulated, whereas TGF-β receptor signaling, WNT signaling, AP-1 transcription factor networking, NF-κB signaling, and PI3K/Akt/mTOR pathways were down-regulated in response to curcumin treatment (Figure 4A). On the other hand, in metastatic C4-2B cells, the signaling pathways of IL6, PTEN-dependent cell cycle arrest and apoptosis and cell cycle: g2/mcheck point pathways were up-regulated, whereas TGF-β receptor signaling, WNT signaling, and the FOXM1 transcription factor network were suppressed by curcumin treatment (Figure 4B). Of note, TGF-β receptor signaling pathway was found to be down-regulated in both less aggressive LNCaP and highly metastatic C4-2B cells over 48 h treatment. 

### 2.6. Comparison of Differentially Regulated Genes in LNCaP and C4-2B at 48 h

**Figure 5:**
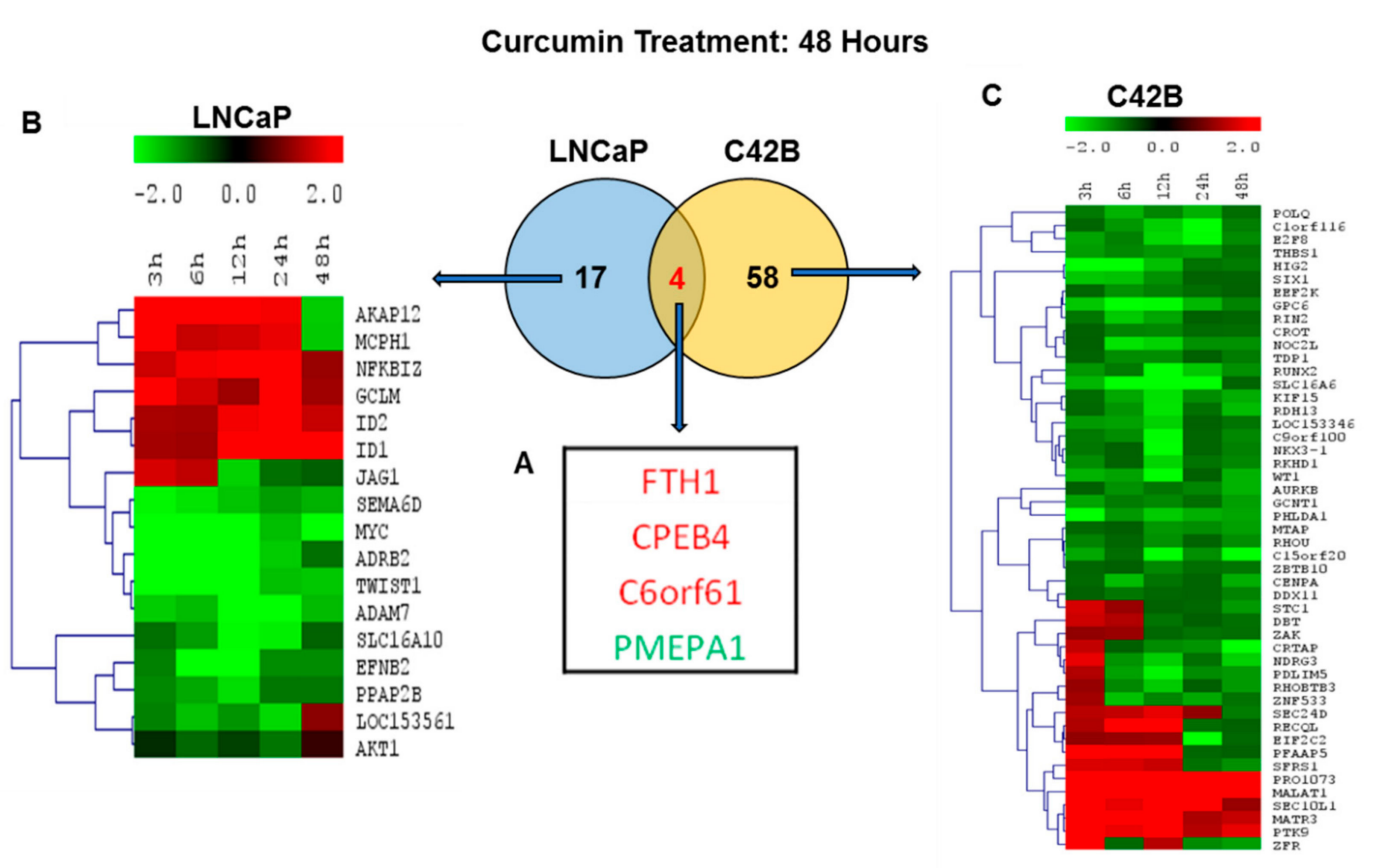
Comparison of differentially regulated genes in LNCaP and C4-2B at 48 h after curcumin treatment. (**A**) Common and unique gene expression changes in LNCaP and C4-2B cell lines. Venn diagram showing the overlap of (four genes) genes in both the LNCaP (blue) and C4-2B (yellow) cells with two-fold expression cut-off at 48 h. Heatmap of top up-regulated and down-regulated genes in (**B**) Hierarchical cluster showing the expression levels of 17 significant genes unique to LNCaP (blue) and (**C**) 58 genes unique to C4-2B (yellow) cells were clustered into two groups: up-regulated and down-regulated. Expression values are color coded (red= upregulation; green = downregulation).

Further, we analyzed the curcumin gene response in LNCaP and C4-2B cells at a 48 h time point to assess the long-term effect of curcumin treatment with respect to androgen responsiveness and non-responsiveness in less aggressive to metastatic prostate cancer. Interestingly, at 48 h post curcumin treatment, only four genes, *FTH1*, *CPEB4*, *C6orf61*, and *PMEPA1* were found to be modulated in both the LNCaP and C4-2B cells, (Figure 5A). The genes coded in red color (*FTH1*, *CPEB4*, *C6orf61*) were up-regulated, and the *PMEPA1* gene coded in green was down regulated (Figure 5A). On the other hand, a total of 17 genes were differentially regulated in LNCaP cells and 58 genes in C4-2B cells. The heat map showed here in Figure 5B for LNCaP cells and 5C for C4-2B cells representing the expression levels of up- and down-regulated genes at a –2.0 to 2.0 scale in LNCaP and C4-2B cells at different time points. 

## 3. Discussion

Despite profound advances in prostate cancer therapy, the incidence and mortality rates of prostate cancer have not declined. Until recently, the treatment for late-stage castration-resistant and metastatic prostate cancers was still a main challenge. The major limitation of targeted therapies is that prostate cancer cells eventually develop resistance to them. The combination of targeted drugs/therapies with traditional herbal compounds could provide an alternative solution to this problem with higher anti-cancer efficacy and affordability [22]. Nutraceuticals have been one newer promising therapeutic agent for the earlier prevention of prostate cancer and treatment of aggressive diseases. Curcumin was one of the nutraceuticals which was shown to reduce the risk of cancer progression. To further understand the chemo-preventive potential of curcumin in prostate cancer, we evaluated curcumin associated gene expression responses in androgen-dependent (hormone responsive, LNCaP) and androgen-independent (hormone non-responsive highly metastatic C4-2B) prostate cancer cell lines by Affymetrix oligonucleotide gene chip microarray. Time-based gene expression analysis showed co-regulation of genes involved in specific biochemical pathways in the functional classification of curcumin-associated gene expression response. Hierarchical clustering revealed that the most significant impacts of curcumin happened at 12 h post treatment (1273 up-regulated genes and 1193 down-regulated genes in LNCaP cells, 1682 up-regulated genes and 943 down-regulated genes in C4-2B cells), consistent with our earlier findings that the prostate cancer cell growth inhibitory effects of curcumin was dose and time dependent [23]. In contrast, the lowest number of up and down regulated genes were found at a 48 h time point after curcumin treatment, indicating the long-term effect of curcumin on these genes. A total of 31 genes were commonly affected in both LNCaP and C4-2B cells. Venn diagram analysis of differentially expressed genes confirmed there was only 10% of impacted genes shared between LNCaP and C4-2B cells, which was consistent with disparities in hormone responsiveness and aggressiveness within these two prostate cancer cell lines. 

*MYC*, *ATF3*, *PMEPA1*, *AURKB*, and *HMOX-1* were identified as novel targets of curcumin in both less aggressive and highly aggressive metastatic prostate cancer cells in our study. The *MYC* gene, which is located on human chromosome 8, is a proto-oncogene required for many functions, including apoptosis, cell cycle, differentiation and growth [24]. Consistent with previous reports [25,26], the *MYC* pro-oncogene was found to as a central pathway node with the highest number of interacting genes which are inhibited by curcumin treatment in prostate cancer cells. The data suggests more integrated *MYC* function in both LNCaP cells as well as more aggressive C4-2B cells, which also may provide a platform for evaluating tumorigenicity and defects in MYC response pathways. It has been shown that androgen signaling alone or in combination with other signaling pathways play an important role in the development of prostate cancer, hormone resistance as wells as relapse of the disease [27,28]. The antagonistic relationship at the gene expression level was observed between AR and MYC in prostate cancer context [29]. *MYC* overexpression was shown to deregulate the AR transcriptional program to drive prostate tumorigenesis. Our findings showed first time showed that *MYC* is a direct target of curcumin in the context of both less aggressive and highly aggressive metastatic prostate cancer, further confirming the chemo-preventive and therapeutic potential curcumin in prostate cancer. 

The stress response mediator, *ATF3*, was frequently down-regulated in prostate cancer. However, our study showed that *ATF3* was up-regulated by curcumin in both LNCaP and C4-2B cells. Various oncogenic pathways were reported to suppress the expression of *ATF3,* including androgen receptor signaling [30]. Wang and Zang provide the genetic evidence supporting the role of ATF3 as a tumor suppressor in a subset of prostate cancers with PTEN dysfunction [30]. Aurora kinases, which is overexpressed in prostate cancer patients [31], was found to be down-regulated by curcumin treatment in both androgen-dependent and independent stages. *AURKB*, in combination with *EGFR* knockdown, have shown enhanced therapeutic effect by inhibiting PC3 cell proliferation and inducing apoptosis in vitro, whereas androgen-dependent cancer cells, LNCaP, remain unaffected by the endogenous expression levels [32]. The dysregulation of both androgen and TGF-β signaling play a key role in prostate tumorigenesis and promote pro-metastatic gene expression and bone metastases in a mouse prostate cancer model [33]. *PMEPA1* gene has been established as an androgen/TGF-β responsive gene modulating both androgen and TGF-β signaling via similar negatively regulated feedback loop [33,34]. Here, we reported that the expression of *PMEPA1* was down-regulated in both LNCaP and C4-2B cells by curcumin as a result of the inhibition of TGF-β and androgen signaling pathways. Our lab further identified *PMEPA1* isoforms (a, b, and d) with distinct functions to regulate androgen and TGF-β signaling and different expression pattern in androgen-dependent and independent prostate cancer cells (unpublished data). The impacts of curcumin treatment on these isoforms need to be further clarified. The expression of *HMOX-1* was reported to be enhanced in cancer cells. Moreover, anti-cancer therapies, including chemo-, radio-, and photodynamic therapy, further induced its expressions, which blocked the treatment effectiveness [35]. *HMOX-1* influences tumor initiation and progression in part by alternating E-cadherin expression from tumor-associated macrophages [36]. In contrast, *HMOX-1* inhibits the migration and growth of prostate cancer cells by modulating the architecture of cell-cell interactions [37,38]. Our data confirmed the tumor-inhibitory effects of *HMOX-1* gene in prostate cancer cells, which was up-regulated by curcumin treatment.

Further, evaluation of significantly regulated top Canonical pathways in LNCaP and C4-2B cells showed that Wnt, PIK3/AKT/mTOR, and NF-κB signaling pathways were primarily inhibited by curcumin treatment, and PTEN dependent cell cycle arrest and apoptosis pathways were found to be elevated. Several preclinical studies using prostate cancer models showed that curcumin modulated the androgen receptor signaling and downstream targets such as *VEGF*, *PTEN*, and *NF-κB* [29]. Our findings were consistent with previous reports that the dysregulation of these signaling pathways contribute to prostate cancer initiation and progression [23]. Of note, the TGF-β signaling pathway was inhibited by curcumin treatment in androgen-dependent and independent manners. It has been reported that curcumin inhibits TGF-β signaling in non-prostate solid tumors, including cancer of cervical, breast, and pancreas, by perturbing Wnt/β-catenin signaling pathways as well as subsequent tumor growth and migration [30,31,39]. Curcumin was also shown to induce *PPAR-γ* gene expression and inhibit hepatic stellate cell (HSC) activation by interrupting TGF-β signaling in vitro [40]. In addition, the therapeutic effect of curcumin on colon carcinogenesis induced by activation of TGF-β signaling lead to novel and more effective treatments for colon cancer [41]. Aberrant activation of TGF-β signaling has shown to play a critical role in prostate cancer progression in castration-resistant and metastasis patients. Our data might provide the evidence that curcumin could be used as a therapeutic strategy for advanced prostate cancer.

Gene ontology and network analysis of differentially expressed genes common in LNCaP and C4-2B for long-term effects of curcumin only showed 3 up-regulated genes: Ferritin Heavy Chain 1 (*FTH1*), Cytoplasmic Polyadenylation Element Binding Protein 4 (*CPEB4*), and *C6orf61*, the Minichromosome Maintenance 9 Homologous Recombination Repair Factor (*MCM9*) and one down-regulated gene *PMEPA1*. These genes are known to be regulators of the cancer phenotype. The *FTH1* gene was shown as one major intracellular iron storage protein. Downregulation of *FTH1* resulted in prostate cancer development and progression [42]. We here first time showed that *FTH1* gene is up-regulated by curcumin treatment even after 48 h, consistent with its prostate cancer growth inhibitory functions. The varied expressions of *CPEB* genes were found to associate with tumorigenesis, tumor growth, invasiveness and angiogenesis. It was further shown that *CPEB4* be highly involved in cancer progression. The study from Zeng et al. showed the that *CPEB4* may function as a tumor suppressor in head and neck squamous cell carcinoma, and hypermethylation of the *CPEB4* gene accountable for the downregulation of *CPEB4* expression lead to tumorigenesis of head and neck squamous cell carcinoma [43]. Our study verified the tumor suppressor function of *CPEB4* gene which was up-regulated by curcumin treatment after 48 h in prostate cancer cells. An earlier study has shown that the deletion of *MCM9* produced a functional defect of HR repair in cancer. Epigenetic suppression of *MCM9* predisposes cancer cells to cisplatin sensitivity [44]. Enhanced expressions of all these genes functioning as tumor suppressor accounted for the long-term growth inhibitory effects of curcumin in prostate cancer cells, further suggesting their potential as novel anti-prostate cancer targets. 

## 4. Materials and Methods

### 4.1. Cell Culture and Reagents

**Scheme 1:**
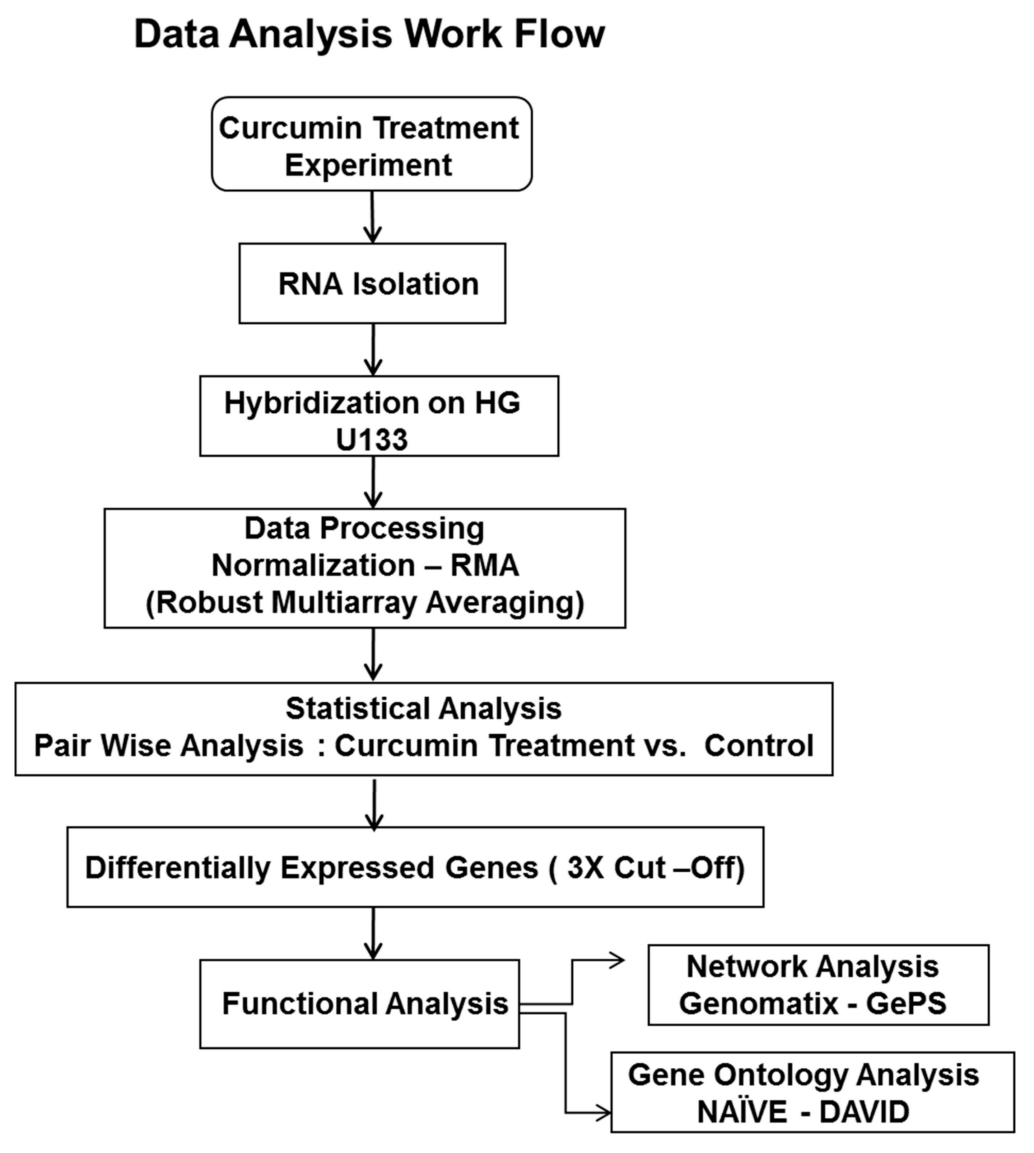
Schematic diagram of microarray bioinformatics data analysis of curcumin associated gene expression responses in early androgen responsive (LNCaP) and late-stage androgen-independent (C4-2B) prostate cancer cells.

The human tumor derived parental androgen-dependent prostate cancer cell line, LNCaP, and androgen-independent metastatic prostate cancer cell line, C4-2B, were obtained from American Type Culture Collection (ATCC), Manassas, VA and were grown in the cell culture medium and conditions recommended by the supplier. The 1.0 × 10^6^ cells were seeded in 10 cm dishes. After 24 h, the cells were treated with curcumin at 10 µM concentration for 3, 6, 12, 24, and 48 h. Curcumin was purchased from LKT Labs (Saint Paul, Minnesota, MN, USA). Control cells were treated with vehicle control, 0.1% DMSO (Sigma-Aldrich, St. Louis, MO, USA). The High-density oligonucleotide human genome GeneChip array HG-U133 was procured from Affymetrix, Santa Clara, CA, USA [22]. The Schematic diagram of bioinformatic analysis of the raw gene expression data output (CEL files) was shown in Scheme 1. The Robust Multi-array Analysis (RMA, http://rmaexpress.bmbolstad.com) and a single-probe analysis approach ChipInspector (Genomatix GmbH, Munich, Germany) was used. ChipInspector software (http://www.genomatix.de) analyzed raw gene expression data at the single probe levels by matching single probes to transcripts, normalizing the total intensities and by the Significance Analysis of Microarrays (SAMs) and enrichment of significantly altered signal intensities. The functional Gene ontology, network and pathway analysis were done by Genomatix-GePS and DAVID software as previously described methods [23,45,46]. 

### 4.2. RNA Extraction, Labeling and Gene Expression Analysis

Total RNA was isolated from the LNCaP and C4-2B cells with and without curcumin treatment with RNeasy Mini Kit (Qiagen, Germantown, MD, USA) following manufacturer’s protocol. The quality of RNA was measured by electrophoresis using 1% agarose formaldehyde gel. The RNA was further quantified by UV-Vis spectrophotometer. The biotin labeled poly (A) RNA samples were hybridized to Affymetrix human genome array GeneChip^®^ HG U133 Plus 2.0 containing probe sets and 38,500 well characterized human genes. The biotylation of poly (A) RNA was carried out by in vitro transcription using MEGA script T7 in vitro Transcription Kit (Ambion, Austin, TX, USA) and further purified by using QIAGEN RNeasy spin columns (Qiagen, Germantown, MD, USA) as per manufacturer’s protocol. The fragmented biotin labeled Poly(A) RNA was hybridized at 42 °C for 16 h and prepared according to previously described methods [23,45]. The hybridized GeneChip arrays were washed, stained and scanned with the HP GeneArray Scanner (Hewlett-Packard, Santa Clara, CA, USA) controlled by GeneChip 3.1 Software (Affymetrix, Thermo Fisher Scientific, Waltham, MA, USA). 

### 4.3. Affymterix Gene Chip Microarray Data Analysis

The GeneChip images were analyzed for the probe intensity captured by using Affymetrix GeneChip^®^ Microarray Analysis Software, version 3.1 and Affymetrix Micro DB and Data Mining Tool version 2.0 (Affymetrix, Thermo Fisher Scientific, Waltham, MA, USA), and Statistica version 4.1 (Stat Soft, Inc., Tulsa, OK, USA). Raw gene expression data from CEL files were processed and analyzed using statistical computing language R (‘affy’ Bioconductor package), background subtraction and normalization were performed using Robust Mutli-array Average (RMA) procedure. Normalized data was exported into Excel and fold changes were calculated by dividing gene expression signal value of curcumin-treated cells with control cells at different time points. Genes with fold change ≥2 and ≥3 were considered to be differentially expressed genes and were subjected to Venn analysis, GePS, and Gene Ontology analysis (NAÏVE-DAVID). The gene network analysis for the selected genes was performed by using Genomatix pathway edition of Bibliosphere (Genomatix GmbH, Munich, Germany, www.genomatix.de). Most highly up-regulated and down-regulated DEGs in LNCaP and C4-2B cell lines were compared and visualized by Heat map analysis using R software as previously described methods [23,45]. 

### 4.4. Gene ontology (GO) and Pathway Analysis

The common curcumin treatment associated genes for both LNCaP and C4-2B prostate cancer cells (*n* = 31 genes; 19 up-regulated and 12 down-regulated) were queried by the Genomatix Pathway System (GePS) that utilizes expert-curated GO information from public and proprietary databases (Genomatix GmbH). In an independent approach, genes were also queried by the Database for Annotation, Visualization and Integrated Discovery (DAVID) software (http://david.abcc.ncifcrf.gov). The Functional Classification Tool was used to assess the functional similarity between input genes [46]. Differentially expressed genes with their corresponding fold change values at different time points were uploaded to Genomatix-GePS for canonical pathway and Gene Ontology analysis. GePS generates interactions between genes based on literature co-citations and the significance of enrichment of genes mapped to different canonical pathways was calculated by the Fischer’s exact test (*p*-value). Color coding in the network is related to the fold changes with red indicating up-regulation, and green indicating down-regulation. Genes with the highest number of interactions occupy the central node in the network and are considered to be significant; this is further evaluated. Canonical pathways and gene ontology terms were ranked by −log (*p*-value). The clustering algorithm used by DAVID classifies highly related genes into functionally related groups.

## 5. Conclusions

Curcumin modulates several signaling pathways and transcription factors by altering their gene expressions or through direct interaction [47]. The biological targets of curcumin include inflammatory cytokines, growth factors, growth factor receptors, enzymes, adhesion molecules, apoptosis-related proteins, and cell cycle proteins. Therefore, curcumin has the potential for the prevention and treatment of various disease including cancers, arthritis, allergies, atherosclerosis, ageing, neurodegenerative disease, hepatic disorders, obesity, diabetes, psoriasis, and autoimmune diseases [47]. Our study highlighted the curcumin-associated gene networking alterations in androgen-dependent non-metastatic prostate cancer LNCaP cells and androgen-independent metastatic prostate cancer C4-2B cells. We undertook a microarray approach for analyzing both long- and short-term effects of curcumin on prostate cancer cells. Our results defined significant genomic alterations in *MYC* pro-oncogene networking, as well as TGF-β signaling—including androgen/TGF-β signaling inhibitor PMEPA1 responding to curcumin treatment in both androgen-dependent and independent prostate cancer cells. Provided the critical roles of *MYC* and TGF-β signaling in the advanced prostate cancer subtypes, our results highly suggest curcumin as a novel anti-cancer supplementary treatment option for non-indolent prostate cancer resistant to hormone and chemical therapies. More clinical trials of curcumin are needed to prove its efficacy in the context of prostate cancer.

In summary, curcumin was shown to suppress the *MYC* pro-oncogene and TGF-β signaling in both an androgen-dependent and independent way. Our data further outlined the major oncogenic signaling pathways inhibited with curcumin treatment as well as tumor inhibitory signaling pathways enhanced by curcumin, highlighting multiple short- and long-term signaling targets of curcumin, which may be applied for novel anti-prostate cancer strategies in both local and advanced hormone independent prostate cancer. 

## Figures and Tables

**Table 1:** Total number of differentially expressed genes in response to curcumin treatment at different time intervals in LNCaP and C4-2B cells.

**LNCaP (>2 Fold Change)**					
	**3 h**	**6 h**	**12 h**	**24 h**	**48 h**
Up-regulated	704	822	1273	806	41
Down-regulated	621	1422	1682	934	119
**C4-2B (>2 Fold Change)**					
	**3 h**	**6 h**	**12 h**	**24 h**	**48 h**
Up-regulated	644	977	1119	410	105
Down-regulated	439	508	943	318	343

## Abbreviations

TGF-β	Transforming growth factor beta
NF-κB	Nuclear factor kappa-light-chain-enhancer of activated B cells
PTEN	Phosphatase and tensin homolog
MAPK	Mitogen-activated protein kinase
EGFR	Epidermal growth factor receptor
AR	Androgen Receptor
TNF	Tumor necrosis factor
COX-2	Cyclooxygenase-2
5-LOX	5-lipoxygenase
TNF	Tumor necrosis factor
IL6	Interleukin 6
KLK3	Kallikrein related peptidase 3
PSA	Prostate-specific antigen
TMPRSS2	Transmembrane serine protease 2
NKX3.1	NK3 Homeobox 1
PMEPA1	Prostate transmembrane protein, androgen induced 1
HMOX1	Heme Oxygenase-1
ATF–3	Cyclic AMP-dependent transcription factor
GePS	Genomatix Pathway System
RAF1	Raf-1 Proto-Oncogene, Serine/Threonine Kinase
BCL6	B-Cell CLL/Lymphoma 6
IGF1R	Insulin-Like Growth Factor 1 Receptor
SMAD7	SMAD Family Member 7
FOXO3	Forkhead Box O3
AKT1	V-Akt Murine Thymoma Viral Oncogene Homolog 1
RAD51	RAD51 Recombinase
SOX4	(SRY (Sex Determining Region Y)-Box 4
WT1	Wilms Tumor 1
E2F2	E2F Transcription Factor 2
MALAT1	Metastasis Associated Lung Adenocarcinoma Transcript 1
FOXM1	Forkhead box M1
EWSR1	Ewing sarcoma breakpoint region 1
SESN2	Sestrin-2
GCLM	Glutamate-Cysteine Ligase Modifier Subunit
AFF4	AF4/FMR2 Family Member 4
ELF3	E74 Like ETS Transcription Factor 3
CREM	CAMP Responsive Element Modulator
BIRC4	Bculoviral IAP repeat-containing protein 4
RIOK3	RIO Kinase 3
PIK3R3	Phosphoinositide-3-Kinase Regulatory Subunit 3
UBE2H	Ubiquitin Conjugating Enzyme E2 H
C6orf62	Chromosome 6 open reading frame 62
DDX11	DEAD/H-Box Helicase 11
CROT	Carnitine O-Octanoyltransferase
HIG2	Hypoxia-inducible gene 2
C1orf116	Chromosome 1 Open Reading Frame 116
DFFB	DNA Fragmentation Factor Subunit Beta
GPC6	Glypican-6
C15orf20	Chromosome 15 Open Reading Frame 20
ADRB2	Adrenoceptor Beta 2
SLC16A6	Solute Carrier Family 16 Member 6
